# Poor Colonoscopy Bowel Prep in Cognitively Impaired 85‐Year‐Old with Low Education: Another Case Example

**DOI:** 10.1002/alz.089102

**Published:** 2025-01-09

**Authors:** Juliana S Burt, Yonah Joffe, Kara Eversole, Faith Kimmet, David Estores, Franchesca Arias, Benjamin A Chapin, Catherine C. Price

**Affiliations:** ^1^ University of Florida, Gainesville, FL USA

## Abstract

**Introduction:**

Colonoscopies are routine procedures performed primarily on adults over the age of 50; however, there is little known about the influence of social determinants of health on successful completion of colonoscopies. Inadequate at‐home bowel preparation can result in increased procedure duration, decreased cancer detection, and may necessitate a repeated colonoscopy, putting undue stress on the patient. Research suggests neurocognitive disorder is a risk factor for poor bowel preparation in older adults; however, lower education may confound neurocognitive findings, independently contributing to risk of incomplete colonoscopies. Research reports patients with a high school diploma were significantly more likely to have inadequate bowel prep compared to patients with college education. The present case study underscores the importance of considering social determinants of health when scheduling patients for colonoscopies.

**Case Presentation Demographics:**

JR is an 85‐year‐old non‐Hispanic Black male referred for preoperative neuropsychological testing prior to a scheduled colonoscopy. He was raised in the rural South and became truant after the 4^th^ grade. He currently lives alone, receiving limited assistance from family.

**Background:**

Five months prior to the scheduled colonoscopy, JR was admitted to hospital due to fecal impaction and rectal pain; he was referred for a colonoscopy to follow up his symptoms. Neuropsychological Testing: revealed 3^rd^ grade reading level and deficits in list learning, delayed recall, and semantic fluency. Problem: The patient arrived disorientated to the purpose of the visit, believing he would undergo the colonoscopy on the day of the neuropsychological evaluation. Consequently, JR completed his bowel preparation the night before and had slept on his bathroom floor. Notably, the procedure was scheduled 13 days later. Outcome: Due to unnecessarily early completion of bowel prep and resultant need for more preparatory medication, the procedure was rescheduled for four weeks later. At the time of this report, JR’s procedure date has not arrived.

**Discussion:**

This case supports research suggesting neurocognitive impairment and lower education are predictive of patients’ poor preoperative planning and bowel preparation. Moreover, this case highlights the need for increased family involvement and attention from providers for patients of advanced age, lower education, and neurocognitive impairment referred for colonoscopies.

